# *exo*-10,11-Dibromo­tricyclo­[6.3.1.0^2,7^]dodeca-2,4,6,9-tetra­ene

**DOI:** 10.1107/S1600536811041730

**Published:** 2011-10-22

**Authors:** Kevin R. D. Johnson, Christopher O. Bender, René T. Boeré

**Affiliations:** a Department of Chemistry and Biochemistry, University of Lethbridge, Lethbridge, AB, Canada T1K 3M4

## Abstract

The title compound, C_12_H_10_Br_2_, is a bridged ring system based on a homobenzonorbornadiene framework. The *exo* configuration of one of the Br atoms was previously assigned *via* NMR correlations and has now been confirmed by the geometry of the solid-state structure. The compound features a Br—C—C—Br torsion angle of 66.68 (12)°, whereby the C atoms in the calculation are respectively *sp* ^3^- and *sp* ^2^-hybridized.

## Related literature

For the structure of a closely related tribromide compound, see: Hökelek *et al.* (1991 ▶). For other similar solid-state structures based on a homobenzonorbornadiene framework, see: Daştan *et al.* (1994 ▶); Balci *et al.* (1996 ▶); Mangion *et al.* (2001 ▶). For synthesis of the title compound, see: Kitahonoki *et al.* (1969 ▶). For derivatization, see: Çakmak & Balci (1989 ▶); Bender *et al.* (2003 ▶).
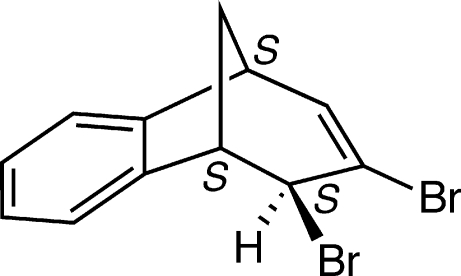


## Experimental

### 

#### Crystal data

C_12_H_10_Br_2_
*M* *_r_* = 314.02
Triclinic, 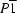

*a* = 6.8554 (5) Å
*b* = 8.0926 (6) Å
*c* = 10.1024 (7) Å
α = 78.936 (1)°
β = 78.867 (1)°
γ = 83.665 (1)°
*V* = 538.13 (7) Å^3^
*Z* = 2
Mo *K*α radiation
μ = 7.49 mm^−1^
*T* = 173 K
0.37 × 0.36 × 0.09 mm

#### Data collection

Bruker APEXII CCD area-detector diffractometer
Absorption correction: multi-scan (*SADABS*; Bruker, 2008 ▶) *T*_min_ = 0.497, *T*_max_ = 0.746
6108 measured reflections
2354 independent reflections
2240 reflections with *I* > 2σ(*I*)
*R*_int_ = 0.013

#### Refinement

*R*[*F* ^2^ > 2σ(*F* ^2^)] = 0.016
*wR*(*F* ^2^) = 0.040
*S* = 1.03
2354 reflections
128 parameters
H-atom parameters constrained
Δρ_max_ = 0.58 e Å^−3^
Δρ_min_ = −0.45 e Å^−3^

### 

Data collection: *APEX2* (Bruker, 2008 ▶); cell refinement: *SAINT-Plus* (Bruker, 2008 ▶); data reduction: *SAINT-Plus*; program(s) used to solve structure: *SHELXS97* (Sheldrick, 2008 ▶); program(s) used to refine structure: *SHELXTL* (Sheldrick, 2008 ▶); molecular graphics: *X-SEED* (Barbour, 2001 ▶); software used to prepare material for publication: *publCIF* (Westrip, 2010 ▶).

## Supplementary Material

Crystal structure: contains datablock(s) I, global. DOI: 10.1107/S1600536811041730/pv2455sup1.cif

Structure factors: contains datablock(s) I. DOI: 10.1107/S1600536811041730/pv2455Isup2.hkl

Supplementary material file. DOI: 10.1107/S1600536811041730/pv2455Isup3.cml

Additional supplementary materials:  crystallographic information; 3D view; checkCIF report

## Figures and Tables

**Fig. 1.**
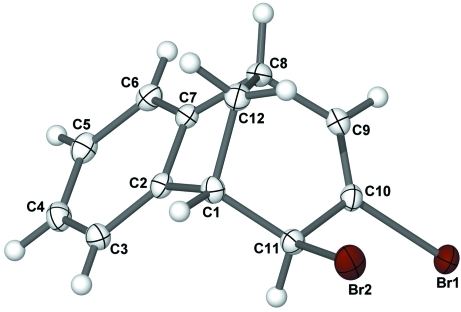
A view of 1 plotted with displacement ellipsoids drawn at the 50% probability level. H atoms are shown as spheres of arbitrary radius.

**Fig. 2.**
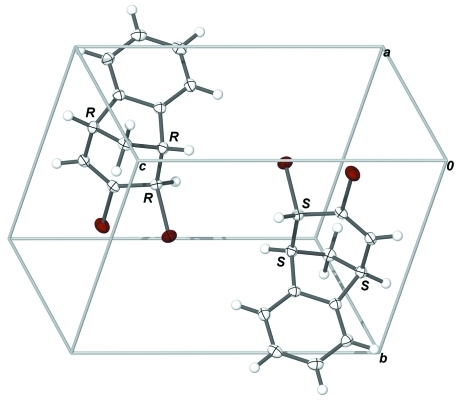
The centrosymmetric pair of enantiomers (SSS and RRR) depicted in a packing diagram.

**Fig. 3.**
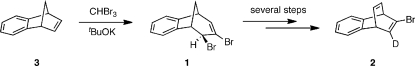
Synthesis of exo-3,4-dibromo-6,7-benzobicyclo[3.2.1]octa-2,6-diene (1) and its conversion to 2-bromo-3-deuteriobenzobarrelene (2).

## supplementary crystallographic information

### Comment

In the title molecule (1) ( Fig. 1), an *exo*-configuration of Br2, which was originally assigned from NMR correlations (Kitahonoki *et al.*, 1969), has now been confirmed by the X-ray data. As such, the stereochemistry of the two enantiomers that assemble in each unit cell can be unambiguously assigned as *RRR* and *SSS* (Fig. 2).

A dihedral angle of 66.7 (1)° was calculated for the Br1—C10—C11—Br2 torsion between the *exo*-configured C11—Br2 and the *sp*^2^ hybridized C10—Br1. Additionally, the compound contains a six-membered ring (C8—C9—C10—C11—C1—C12) that exhibits an interesting envelope-type conformation. This conformation matches that observed in other structures based on a homobenzonorbornadiene framework (Balci *et al.*, 1996). The structure reported by Hokelek *et al.* (1991) differs from 1 only by the presence of a third bromine atom attached to C12 and directed towards the cyclohexene ring (i.e. replacing H12B). However, the precision in C-C bond distances in 1 is on average five times better resulting in a considerably more reliable geometry than that reported for the tribromide.

Compound 1 has proven to be a convenient starting material for the preparation of a variety of substituted benzobarrelenes (Cakmak & Balci, 1989). For example, 1 can be readily converted to 2-bromo-3-deuteriobenzobarrelene (2) over several synthetic steps (Bender *et al.*, 2003) as outlined in Figure 3.

### Experimental

The dibromide (1) was prepared in a one-step process (Kitahonoki *et al.*, 1969) from the addition of dibromocarbene to benzonorbornadiene (3, Figure 3). The desired compound (1, m.p. 355-356 K) was obtained in 27% yield from the reaction mixture by distillation under reduced pressure and recrystallization from acetone-pentane.

### Refinement

Although hydrogen atoms were visible on the Fourier map, they were included at geometrically idealized positions with isotropic displacement parameters and refined in riding mode on their parent atoms with distance constraints: C—H = 1.00, 0.99 and 0.95 Å for methine, methylene and aromatic-type H-atoms, respectively, and *U*_iso_(H) = 1.2*U*_eq_(C). The highest residual peak had a fraction of the electron density of a single H atom and was located 0.77 Å from Br1.

### Crystal data

**Table:**

C_12_H_10_Br_2_	*Z* = 2
*M**_r_* = 314.02	*F*(000) = 304
Triclinic, *P*1	*D*_x_ = 1.938 Mg m^−^^3^
Hall symbol: -P 1	Melting point = 355–356 K
*a* = 6.8554 (5) Å	Mo *K*α radiation, λ = 0.71073 Å
*b* = 8.0926 (6) Å	Cell parameters from 4757 reflections
*c* = 10.1024 (7) Å	θ = 2.6–27.6°
α = 78.936 (1)°	µ = 7.49 mm^−^^1^
β = 78.867 (1)°	*T* = 173 K
γ = 83.665 (1)°	Plate, colourless
*V* = 538.13 (7) Å^3^	0.37 × 0.36 × 0.09 mm

### Data collection

**Table:**

Bruker APEXII CCD area-detector diffractometer	2354 independent reflections
Radiation source: fine-focus sealed tube	2240 reflections with *I* > 2σ(*I*)
graphite	*R*_int_ = 0.013
Detector resolution: 66.06 pixels mm^-1^	θ_max_ = 27.1°, θ_min_ = 2.1°
φ and ω scans	*h* = −8→8
Absorption correction: multi-scan (*SADABS*; Bruker, 2008)	*k* = −10→10
*T*_min_ = 0.497, *T*_max_ = 0.746	*l* = −12→12
6108 measured reflections	

### Refinement

**Table:**

Refinement on *F*^2^	Secondary atom site location: difference Fourier map
Least-squares matrix: full	Hydrogen site location: inferred from neighbouring sites
*R*[*F*^2^ > 2σ(*F*^2^)] = 0.016	H-atom parameters constrained
*wR*(*F*^2^) = 0.040	*w* = 1/[σ^2^(*F*_o_^2^) + (0.0181*P*)^2^ + 0.3469*P*] where *P* = (*F*_o_^2^ + 2*F*_c_^2^)/3
*S* = 1.03	(Δ/σ)_max_ = 0.001
2354 reflections	Δρ_max_ = 0.58 e Å^−^^3^
128 parameters	Δρ_min_ = −0.45 e Å^−^^3^
0 restraints	Extinction correction: *SHELXTL* (Sheldrick, 2008), Fc^*^=kFc[1+0.001xFc^2^λ^3^/sin(2θ)]^-1/4^
Primary atom site location: heavy-atom method	Extinction coefficient: 0.0205 (9)

### Special details

**Table:**

Experimental. A crystal coated in Paratone (TM) oil was mounted on the end of a thin glass fiber and cooled in the gas stream of the diffractometer Kryoflex low temperature device.
Geometry. All e.s.d.'s (except the e.s.d. in the dihedral angle between two l.s. planes) are estimated using the full covariance matrix. The cell e.s.d.'s are taken into account individually in the estimation of e.s.d.'s in distances, angles and torsion angles; correlations between e.s.d.'s in cell parameters are only used when they are defined by crystal symmetry. An approximate (isotropic) treatment of cell e.s.d.'s is used for estimating e.s.d.'s involving l.s. planes.
Refinement. Refinement of *F*^2^ against ALL reflections. The weighted *R*-factor *wR* and goodness of fit *S* are based on *F*^2^, conventional *R*-factors *R* are based on *F*, with *F* set to zero for negative *F*^2^. The threshold expression of *F*^2^ > σ(*F*^2^) is used only for calculating *R*-factors(gt) *etc*. and is not relevant to the choice of reflections for refinement. *R*-factors based on *F*^2^ are statistically about twice as large as those based on *F*, and *R*- factors based on ALL data will be even larger.

### Fractional atomic coordinates and isotropic or equivalent isotropic displacement parameters (Å^2^)

**Table:**

	*x*	*y*	*z*	*U*_iso_*/*U*_eq_	
Br1	0.21938 (3)	0.19786 (2)	0.215988 (17)	0.02899 (7)	
Br2	0.61510 (3)	0.37752 (2)	0.314452 (18)	0.03298 (7)	
C10	0.2653 (2)	0.43063 (19)	0.19221 (16)	0.0202 (3)	
C9	0.2194 (2)	0.5361 (2)	0.08314 (16)	0.0210 (3)	
H9	0.1728	0.4944	0.0141	0.025*	
C11	0.3411 (2)	0.48460 (19)	0.30557 (16)	0.0206 (3)	
H11	0.2516	0.4464	0.3944	0.025*	
C7	0.0743 (2)	0.79247 (18)	0.17148 (15)	0.0193 (3)	
C1	0.3474 (2)	0.67785 (19)	0.28318 (16)	0.0198 (3)	
H1	0.4275	0.7135	0.3443	0.024*	
C2	0.1358 (2)	0.76059 (18)	0.29894 (15)	0.0188 (3)	
C12	0.4241 (2)	0.7434 (2)	0.13037 (16)	0.0224 (3)	
H12A	0.4561	0.8627	0.1142	0.027*	
H12B	0.5428	0.6735	0.0938	0.027*	
C8	0.2415 (2)	0.72350 (19)	0.06857 (16)	0.0209 (3)	
H8	0.2463	0.7860	−0.0274	0.025*	
C5	−0.2360 (2)	0.9194 (2)	0.27206 (18)	0.0246 (3)	
H5	−0.3634	0.9756	0.2636	0.029*	
C3	0.0114 (2)	0.8056 (2)	0.41336 (16)	0.0224 (3)	
H3	0.0520	0.7817	0.5002	0.027*	
C4	−0.1757 (2)	0.8871 (2)	0.39833 (17)	0.0254 (3)	
H4	−0.2624	0.9208	0.4755	0.030*	
C6	−0.1118 (2)	0.8703 (2)	0.15684 (17)	0.0225 (3)	
H6	−0.1544	0.8901	0.0707	0.027*	

### Atomic displacement parameters (Å^2^)

**Table:**

	*U*^11^	*U*^22^	*U*^33^	*U*^12^	*U*^13^	*U*^23^
Br1	0.04010 (11)	0.01927 (9)	0.02778 (10)	−0.00622 (7)	−0.00221 (7)	−0.00608 (6)
Br2	0.03114 (11)	0.03503 (11)	0.03414 (11)	0.01206 (7)	−0.01449 (7)	−0.00890 (7)
C10	0.0213 (7)	0.0171 (7)	0.0224 (7)	−0.0028 (6)	−0.0008 (6)	−0.0064 (6)
C9	0.0197 (7)	0.0237 (8)	0.0207 (7)	−0.0012 (6)	−0.0027 (6)	−0.0078 (6)
C11	0.0213 (7)	0.0206 (7)	0.0197 (7)	0.0022 (6)	−0.0047 (6)	−0.0040 (6)
C7	0.0222 (7)	0.0155 (7)	0.0200 (7)	−0.0026 (6)	−0.0032 (6)	−0.0028 (5)
C1	0.0188 (7)	0.0206 (7)	0.0214 (7)	−0.0012 (6)	−0.0047 (6)	−0.0057 (6)
C2	0.0197 (7)	0.0156 (7)	0.0216 (7)	−0.0022 (5)	−0.0037 (6)	−0.0036 (5)
C12	0.0204 (7)	0.0217 (8)	0.0241 (8)	−0.0029 (6)	−0.0003 (6)	−0.0041 (6)
C8	0.0233 (8)	0.0206 (7)	0.0176 (7)	−0.0005 (6)	−0.0026 (6)	−0.0021 (6)
C5	0.0189 (7)	0.0211 (8)	0.0334 (9)	−0.0001 (6)	−0.0045 (6)	−0.0048 (6)
C3	0.0254 (8)	0.0218 (8)	0.0206 (7)	−0.0027 (6)	−0.0038 (6)	−0.0052 (6)
C4	0.0239 (8)	0.0240 (8)	0.0270 (8)	−0.0019 (6)	0.0022 (6)	−0.0079 (6)
C6	0.0234 (8)	0.0199 (7)	0.0250 (8)	−0.0019 (6)	−0.0075 (6)	−0.0026 (6)

### Geometric parameters (Å, °)

**Table:**

Br1—C10	1.9061 (15)	C1—H1	1.0000
Br2—C11	1.9908 (15)	C2—C3	1.382 (2)
C10—C9	1.325 (2)	C12—C8	1.539 (2)
C10—C11	1.498 (2)	C12—H12A	0.9900
C9—C8	1.517 (2)	C12—H12B	0.9900
C9—H9	0.9500	C8—H8	1.0000
C11—C1	1.541 (2)	C5—C4	1.385 (2)
C11—H11	1.0000	C5—C6	1.399 (2)
C7—C6	1.380 (2)	C5—H5	0.9500
C7—C2	1.400 (2)	C3—C4	1.397 (2)
C7—C8	1.526 (2)	C3—H3	0.9500
C1—C2	1.521 (2)	C4—H4	0.9500
C1—C12	1.541 (2)	C6—H6	0.9500
			
C9—C10—C11	123.77 (14)	C8—C12—C1	100.48 (12)
C9—C10—Br1	119.62 (12)	C8—C12—H12A	111.7
C11—C10—Br1	116.49 (11)	C1—C12—H12A	111.7
C10—C9—C8	119.94 (14)	C8—C12—H12B	111.7
C10—C9—H9	120.0	C1—C12—H12B	111.7
C8—C9—H9	120.0	H12A—C12—H12B	109.4
C10—C11—C1	111.60 (13)	C9—C8—C7	107.28 (12)
C10—C11—Br2	109.24 (10)	C9—C8—C12	107.79 (13)
C1—C11—Br2	108.86 (10)	C7—C8—C12	100.49 (12)
C10—C11—H11	109.0	C9—C8—H8	113.4
C1—C11—H11	109.0	C7—C8—H8	113.4
Br2—C11—H11	109.0	C12—C8—H8	113.4
C6—C7—C2	120.87 (14)	C4—C5—C6	120.84 (15)
C6—C7—C8	131.24 (14)	C4—C5—H5	119.6
C2—C7—C8	107.87 (13)	C6—C5—H5	119.6
C2—C1—C11	109.58 (12)	C2—C3—C4	118.46 (15)
C2—C1—C12	100.65 (12)	C2—C3—H3	120.8
C11—C1—C12	108.97 (12)	C4—C3—H3	120.8
C2—C1—H1	112.3	C5—C4—C3	120.70 (15)
C11—C1—H1	112.3	C5—C4—H4	119.7
C12—C1—H1	112.3	C3—C4—H4	119.7
C3—C2—C7	120.78 (14)	C7—C6—C5	118.33 (15)
C3—C2—C1	130.10 (14)	C7—C6—H6	120.8
C7—C2—C1	109.11 (13)	C5—C6—H6	120.8
			
C11—C10—C9—C8	−0.4 (2)	C2—C1—C12—C8	−42.36 (14)
Br1—C10—C9—C8	175.59 (11)	C11—C1—C12—C8	72.80 (14)
C9—C10—C11—C1	3.2 (2)	C10—C9—C8—C7	−72.51 (18)
Br1—C10—C11—C1	−172.90 (10)	C10—C9—C8—C12	34.95 (19)
C9—C10—C11—Br2	−117.19 (15)	C6—C7—C8—C9	−95.45 (19)
Br1—C10—C11—Br2	66.68 (12)	C2—C7—C8—C9	82.96 (15)
C10—C11—C1—C2	68.38 (16)	C6—C7—C8—C12	152.03 (16)
Br2—C11—C1—C2	−170.97 (10)	C2—C7—C8—C12	−29.56 (15)
C10—C11—C1—C12	−40.88 (17)	C1—C12—C8—C9	−68.19 (14)
Br2—C11—C1—C12	79.77 (13)	C1—C12—C8—C7	43.94 (14)
C6—C7—C2—C3	0.3 (2)	C7—C2—C3—C4	−1.4 (2)
C8—C7—C2—C3	−178.29 (14)	C1—C2—C3—C4	177.45 (15)
C6—C7—C2—C1	−178.77 (14)	C6—C5—C4—C3	0.4 (2)
C8—C7—C2—C1	2.62 (16)	C2—C3—C4—C5	1.1 (2)
C11—C1—C2—C3	91.68 (19)	C2—C7—C6—C5	1.1 (2)
C12—C1—C2—C3	−153.62 (16)	C8—C7—C6—C5	179.37 (15)
C11—C1—C2—C7	−89.35 (15)	C4—C5—C6—C7	−1.5 (2)
C12—C1—C2—C7	25.36 (15)		
